# Real-time luminescence enables continuous drug–response analysis in adherent and suspension cell lines

**DOI:** 10.1080/15384047.2022.2065182

**Published:** 2022-04-21

**Authors:** Clayton M. Wandishin, Charles John Robbins, Darren R. Tyson, Leonard A. Harris, Vito Quaranta

**Affiliations:** a Department of Biochemistry, Vanderbilt University, Nashville, TN USA; b Department of Biochemistry, Vanderbilt University School of Medicine, Nashville, TN USA; c Department of Biomedical Engineering, University of Arkansas, Fayetteville, AR USA

**Keywords:** DIP rate, continuous assay, cell viability, suspension cells, drug response, quantitative analysis, drug screening, real-time luminescence

## Abstract

The drug-induced proliferation (DIP) rate is a metric of *in vitro* drug response that avoids inherent biases in commonly used metrics such as 72 h viability. However, DIP rate measurements rely on direct cell counting over time, a laborious task that is subject to numerous challenges, including the need to fluorescently label cells and automatically segment nuclei. Moreover, it is incredibly difficult to directly count cells and accurately measure DIP rates for cell populations in suspension. As an alternative, we use real-time luminescence measurements derived from the cellular activity of NAD(P)H oxidoreductase to efficiently estimate drug response in both adherent and suspension cell populations to a panel of known anticancer agents. For the adherent cell lines, we collect both luminescence reads and direct cell counts over time simultaneously to assess their congruency. Our results demonstrate that the proposed approach significantly speeds up data collection, avoids the need for cellular labels and image segmentation, and opens the door to significant advances in high-throughput screening of anticancer drugs.

## Graphical abstract

**Figure:**
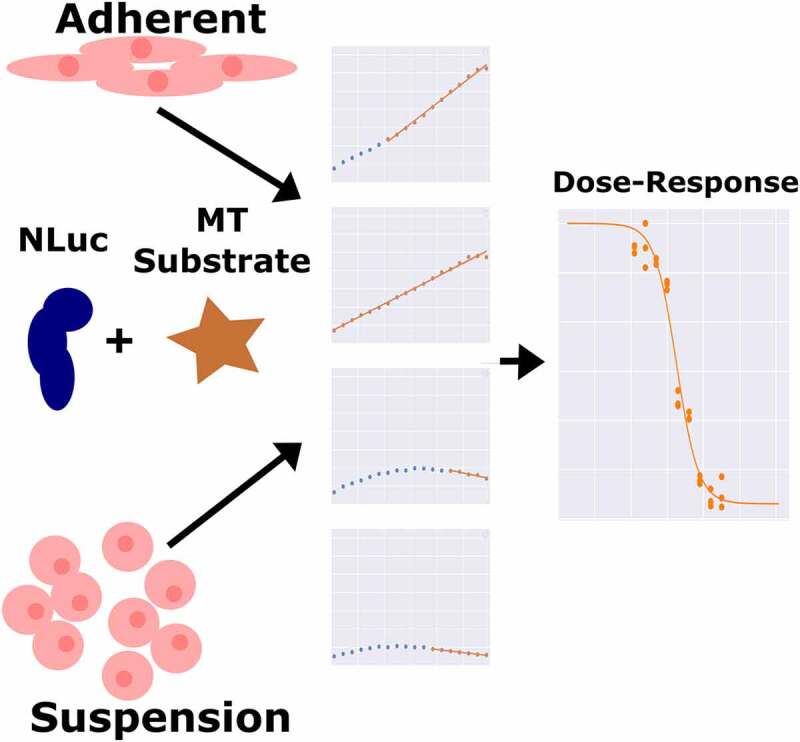

## Introduction

Assessing cellular drug response across multiple cell lines and types is an integral component of modern cancer research. This is primarily done by taking a single cellular viability^1^ measurement before and after the addition of a drug across a range of concentrations in what is known as a “fixed-endpoint assay”. These measurements are then used to produce a dose–response curve to assess efficacy and potency. However, fixed endpoint assays contain a multitude of inherent biases such as the time delay effect (slow-acting drug bias), seeding density variability (T_0_), exponential growth vs. percent viability (ratio changes based on how far out the endpoint is taken), cellular growth rate dependence, and the lack of ability to produce negative values (minimum efficacy of zero) that can result in inaccurate determinations of both efficacy and potency in a variety of scenarios, potentially mischaracterizing both effective and ineffective treatments.^1^ A more robust alternative is to assess viability via a continuous metric. Continuous viability assays have gained substantial interest in the scientific community as they overcome the biases associated with a fixed endpoint and provide a more detailed representation of cellular drug response over time. Continuous viability assays are conducted by taking intermediate measurements across a given time interval, with short measurement intervals and extended time courses giving the most detailed information. While fixed-endpoint data yields a single number that can easily be used in dose–response curve generation, continuous assays generate multiple values, and thus require derivation to distill responses across a time period down to a single value. Assays such as EZ-MTT address this most simply by taking the slope of the dataset for dose–response curve generation, while alternative approaches such as the GR (growth inhibition rate) metrics and DIP rate address it by expressing each individual data series as a ratio of the basal response.^1–3^ Continuous assays also have their own experimental hurdles that have prevented widespread adoption of the platform, such as requiring a live cell fluorescent label (direct cell counting), inefficient cell segmentation algorithms, and an inability to work well with suspension cell lines (limited by imaging ability).

**Figure 1.**
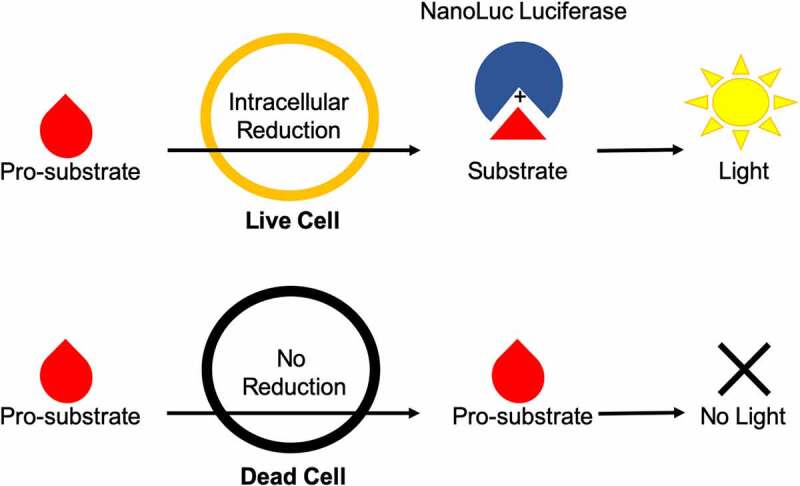
Diagram of Real-Time Luminescence Dynamics. Pro-substrate added to the culture media is rapidly metabolized by live cells via intracellular reduction into active substrate. The active substrate then reacts with NanoLuc luciferase to produce light. Dead cells are not able to metabolize the pro-substrate and therefore do not contribute to the amount of active substrate produced and subsequent light generation within the assay.

**Figure 2.**
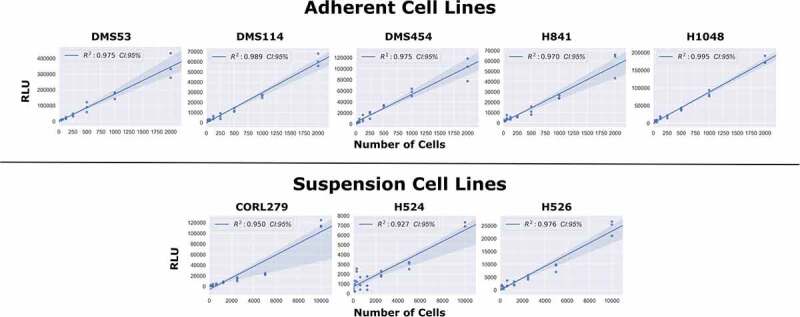
Comparison of Static Luminescent Signal and Cell Count. A range of cell lines were serially diluted by a factor of 2 from either 10,000 cells (suspension lines) or 2,000 cells (adherent lines). Assay reagents were then added to the wells and the plate was allowed to equilibrate for 1 hour. The luminescence measurements were then obtained, with the above graph showing the regression values among the static measurements of luminescence compared to varying cell seeding densities.

Recently, a new continuous luminescence-based viability assay has been developed that indirectly measures the cellular reductive capacity through metabolic conversion of a pro-substrate to substrate (Figure 1). The novel low-toxicity and membrane-permeable NanoLuc luciferase pro-substrate rapidly diffuses into cells and is converted to active substrate (furimazine) primarily by NAD(P)H oxidoreductase, a ubiquitous and established enzyme in the cellular metabolic process.^4–12^ Once the substrate is generated, binding to the luciferase and subsequent enzymatic cleavage produces luminescence. These luminescence values correlate well with cell counts in static measurements (Figure 2) suggesting that this system could also be used for continuous luminescence measurements as an alternative to obtaining proliferation rates by direct cell counting. This is especially promising for suspension cell cultures, where direct cell counting is often not a feasible option. Here, we show that by modifying and optimizing the commercial assay protocol for single reagent-addition, the continuous luminescence data can be used as an alternative for direct cell counting measurements. Briefly, by focusing on the rate of luminescence change in drugged cell conditions and normalizing to the basal rate of change in an undrugged population, the continuous luminescence data can be reduced to a single value, reflecting the expansion and contraction of the cell population in response to drug. This streamlines the quantification of the response to the level of a fixed-endpoint assay, while remaining continuous in origin.^1,11,13,14^ Furthermore, we addressed challenges in the data interpretation by developing a freely available open-source analytical process (coding algorithm). Overall, using continuous luminescence to measure cellular drug response allows quantification regardless of cells being in suspension or adherent culture.

Alt Text: A cartoon diagram depicting how the NanoLuc Real-Time luminescence assay works. There are two flowcharts moving from left to right, above and below each other. The upper flowchart depicts the dynamics of the assay with live cells and the lower flowchart depicts the dynamics with dead cells. Pro substrate is depicted as a red tear drop, substrate is depicted as a red triangle, NanoLuc luciferase is depicted as a blue circle with a wedge missing, cells are depicted as large circles, and light is depicted as a yellow sun.

Alt Text: There are two rows of graphs where the Y-axis is RLU (Relative Luminescence Units) and the X-axis is number of cells. The top row is five adherent cell lines and the bottom row is three suspension cell lines. All of the graphs show a highly linearly correlated regression line with R2 values greater than 0.92 in all cases.

1. Cellular viability is referred to herein as the amount of live, viable, cells within a well.

## Results

### Optimizing the commercial assay for single reagent-addition continuous experiments

In order to utilize the commercial NanoLuc luciferase assay for continuous experiments, we adjusted the supplied protocol. After testing of a variety of conditions addressing NanoLuc enzyme concentration, MT substrate (NanoLuc pro-substrate) concentration, solubilization temperature and duration, cell seeding density, and confluency of culture prior to experimentation (data not shown), the following tenets were obtained. First and foremost, the optimal reagent preparation was found to be 20 µL of both the NanoLuc enzyme (1000X supplied) and the MT substrate (1000X supplied) dissolved in to 25 mL of culture medium supplemented with 10% FBS (Fetal Bovine Serum). We found the solubility of the MT substrate specifically, to be highly dependent on temperature.

During optimization, it was observed that the assay was more sensitive to temperature fluctuations during reads than previously anticipated. In order to address this, travel time between the plate incubator and reader was reduced to a minimum, and an additional incubation delay within a pre-warmed reader was added. The resulting optimized protocol based on these findings is available at https://github.com/QuLab-VU/RT-Glow/tree/master/RT-Glo%20Paper.

### Comparing luminescence to direct cell counts in proliferating cell populations

We first confirmed the relationship between luminescence signal and cell number by comparing luminescence readings and direct cell counts in cultured wells with predefined numbers of cells (Figure 2, and see Methods). To this end, we took luminescence reads across serially diluted cell concentrations after addition of assay reagents followed by 1 h of equilibration. These static, single time-point measurements revealed a strong linear correlation between luminescence signal intensity and cell number in five adherent and three suspension cell lines (Figure 2). These results suggested that it is possible to monitor cell proliferation via luminescence in continuous culture over time, as a substitute for the more laborious direct cell count sampling.

**Figure 3.**
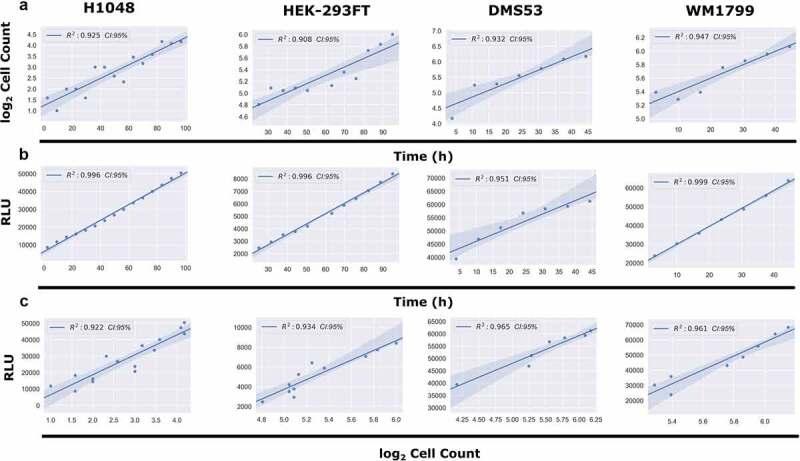
Comparison of Continuous Luminescent Signal and Cell Counts Over Time. (A) Comparison of the log_2_ transformed cell counts over time in four adherent cell lines. Cell counts were log_2_ transformed in order to linearize the data for subsequent comparisons. (B) Comparison of the continuous luminescent signal over time for the same four adherent lines from panel A. (C) Comparison of the correlation between continuous luminescent signal and log_2_ transformed cell count over time using a best-fit linear regression model. All conditions show R^2^ correlation coefficients >0.92.

To test the feasibility of continuous luminescence as an alternative for direct cell counting, we cultured multiple adherent cell lines (see Methods) and took both luminescence and direct cell counts every 4 h for 100 h (Figure 3). Proliferation rates were then generated by taking the slope of both the raw luminescence and log transformed direct cell counting values and compared (Figure 3). The coefficient of determination (R^2^) between the two proliferation rates was found to be greater than 0.92 in each.

Alt Text: There are four columns of graphs for cell lines (H1048, HEK-293FT, DMS53, and WM1799). Each column is divided into three rows A, B, and C. Row A has graphs with a Y-axis of log2 cell count and an X-axis of time in hours. Each graph shows a best fit linear regression line for the two axes. Row B has graphs with a Y-axis of RLU (Relative Luminescence Units) and an X-axis of time in hours. Each graph shows a best fit linear regression line for the two axes. Row C has graphs with a Y-axis of RLU and an X-axis of log2 cell count. Each graph shows a best fit linear regression line for the two axes.

**Figure 4.**
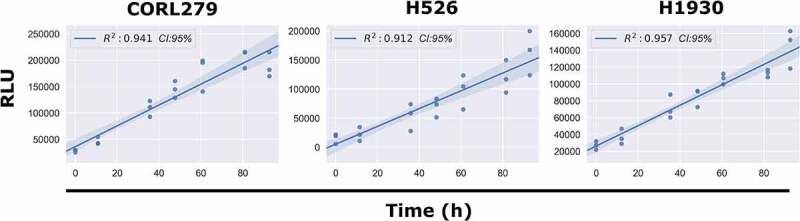
Continuous Luminescence of Suspension Cell Lines. A best-fit linear regression model of continuous luminescence in all suspension cell lines tested shows that minimum luminescent linearity requirements (R^2^ >0.90) are met. Real-time luminescent signal maintains a sufficient linearity for the duration of the assay.

Next, we took continuous luminescence measurements on suspension cell lines, where direct cell counting is not available, to assess if their luminescence remained linear for the duration of the experiment. Since linearity of luminescence signal is a requirement for straightforward analysis of continuous luminescence measurements (taking the slope) it was necessary to confirm this prior to using it as a metric for cell proliferation (see Methods, Determining Linear Assay Range). All three of the suspension lines tested (CORL279, H526, H1930) satisfied this requirement (Figure 4). Taken together, these results from both adherent and suspension cell cultures indicate that continuous luminescent measurements are a viable alternative to direct cell counting to assess cell proliferation over time.

Alt Text: There is a single row of three graphs with RLU (Relative Luminescence Units) on the Y axis and time in hours on the X-axis. The three graphs are for suspension cell lines CORL279, H526, and H1930, respectively. Each graph shows a best fit linear regression line for the two axes.

### Quantifying drug response using continuous luminescence measurements

To explore the usefulness of the assay for continuous measurements of cell proliferation in response to drugs, we treated eight cell lines with several known anticancer agents and cultured them with the assay reagents for 5 d while taking luminescence measurements. Luminescence offers several advantages over conventional cell count assays (see Introduction and Discussion for more details), including speed and ease of execution and analysis for both adherent and suspension cell lines. By combining luminescence with drug-response data, continuous dose–response curves can be rapidly and efficiently generated by quantifying the rate of change in luminescence (slope). Moreover, because luminescence measurements are an indirect quantification of every single cell within a well, the data gleaned from them is much more sensitive and less variable than taking direct cell imaging counts. This is most exemplified when comparing luminescence measurements to direct cell counts produced from imaging only a fraction of a given well (standard practice).

**Figure 5.**
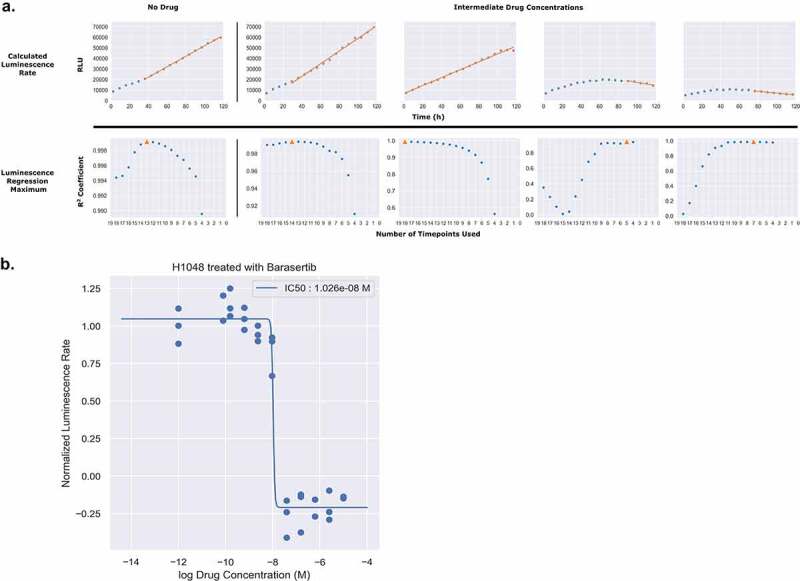
Slicing of Luminescence Data to Obtain Rate. (A) Luminescence rates for each individual drug concentration were calculated by fitting the raw luminescence data to a linear regression model. For each concentration, the number of timepoints used in the regression (slice) was determined by calculating the R^2^ for every possible slicing vector containing more than four points, originating from the end of the assay. The slice producing the maximum R^2^ value is denoted in orange as a triangle. (B) To generate dose–response curves, each of the calculated luminescence rates was normalized to the luminescence rate in the absence of drug and plotted as a normalized rate in respect to the log of the drug concentration. These data were then fitted to a four parameter log logistic function.

**Figure 6.**
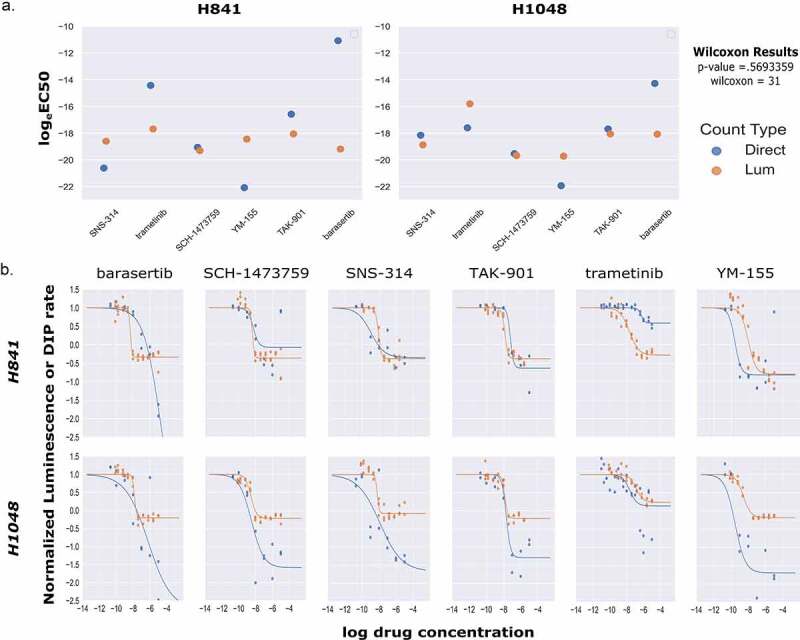
Comparison of EC50 Values and Dose–Response Curve Fits between Luminescent and Direct Cell Counting Measurements. (A) Scatter plot comparison of calculated log_e_EC50 values for both luminescence based and direct cell counting measurements. Across all paired values tested, there was no significant difference between luminescence-based log_e_EC50 values and those obtained from direct cell counting (Wilcoxon signed rank test, p-value = 0.569, W = 31, N = 12). (B) Comparisons of dose–response curves generated by either luminescence (orange) or direct cell counting (blue) for two cell lines across a panel of six drugs.

To generate rates from the continuous luminescence data, we took the slopes of the best fit linear regression lines of the raw luminescence data. An algorithm was developed to compare increasing slices of data points from the end of the assay (defined as peak luminescence in the control condition) by calculating an R^2^ value for each slice, and using the highest R^2^ value’s linear regression slope as the basal rate for which subsequent drug dilution luminescence rates were normalized to. For drugged conditions, a similar process was used, but constrained to the region between the peak luminescence of the drugged condition, and the final timepoint of the assay determined by the peak luminescence of the control (Figure 5A). Once the slopes of the continuous luminescent signals were obtained, they were normalized and plotted against the drug concentration series to obtain dose–response curves (Table 1, Figure 5B). In comparing dose–response curves generated from luminescent or direct cell counting data; overall fitting, data variation, and EC50 values were broadly found to be in agreement (Figure 6). Comparing the EC50 values generated from both luminescence and direct counting measures, a Wilcoxon signed-rank test analysis (nonparametric, paired) generated a Wilcoxon value of 31 and a *p*-value of 0.5693359 (Figure 6A). For the EC50 sample size (N = 12) a Wilcoxon value of 31 exceeds the two-tailed critical value threshold of 13 (α=.05) and we fail to reject the null hypothesis that the EC50 value pairs are not significantly different. Therefore, whether the EC50 was obtained through direct counting measurements or luminescence did not make a significant difference in its value across the cases tested here. To further assess the congruency of dose–response curve generation between luminescence-derived datasets and those from direct counting, the E_max_ values for each cell line and drug pairing were compared based on whether or not the E_max_ values were positive (anti-proliferative/cytostatic [~0]), negative (cytotoxic), or equal to 1 (no drug effect). This is an important observation to make if this type of assay were to be utilized as a first pass drug screening application in order to correctly discern cytotoxic lead compounds from those that are anti-proliferative/cytostatic, or have no effect at all. From 12 paired samples, 11 pairs were found to be in agreement on drug effect mechanism while only one pair (H841/trametinib) was found to lack congruency (Figure 6B). It is also important to note that all of the data used in generating the comparisons in figure 6 were obtained using the same cells within a well for both the luminescence and direct counting measurements. To this end, the analysis being down between the two methods is being generated from the exact same cells, not between equivalents. Across both suspension and adherent cell lines, dose–response curves from luminescence-based rates were generated successfully. The code and associated data are freely accessible in this github repository, “https://github.com/QuLab-VU/RT-Glow/tree/master/RT-Glo%20Paper”.

Alt Text: (A) The diagram consists of two rows, one on top of the other, where the top row has five graphs with a Y-axis of RLU (relative luminescence units) and an X-axis of time in hours. Each of these graphs shows the luminescence data points collected during the experiment, and highlights the slice of data used in determining the luminescence rate. This top row is labeled “Calculated Luminescence Rate”. The bottom row has five corresponding graphs with a Y-axis of R2 coefficient and an X-axis of the number of timepoints used in the luminescence rate calculation. In each graph the number of timepoints used in the final calculation is denoted with an orange triangle. This row is labelled “Luminescence Regression Maximum”. (B) This diagram depicts a dose–response curve of the response of H1048 to Barasertib based on luminescence data. It is an example of the types of dose–response curves that can be generated using continuous luminescence. The overlaid sigmoidal curves fits well, and an IC50 value of 1.026E-08 M is denoted in the top-right corner.

Alt Text: (A) There are two paired scatter plots side by side with a Y-axis of logeEC50 and X-axis labels of SNS-314, trametinib, SCH-1473759, YM-155, TAK-901, and barasertib corresponding to drugs. The scatter plot on the left is for cell line H841 and the one on the right is for cell line H1048. For each X-axis label within each plot, there are a pair of points, one orange denoting the value derived from luminescence, and one blue denoting the value derived from direct counting. Objectively, the majority of these paired points are similar. On the fair right of the diagram are the Wilcoxon signed-rank statistical test results showing a p-value of 0.563359 and a Wilcoxon value of 31. These results indicate that there is not a significant difference in the logeEC50 value between those determined by luminescence and those determined by direct cell counting. (B) There is a panel of 12 dose response curves, two rows of six. The top row is labeled H841 and the bottom row is labeled H1048. Columns 1–6 are labeled barasertib, SCH-1473759, SNS-314, TAK-901, trametinib, and YM-155, respectively. In every graph, the Y-axis is the Normalized Luminescence or DIP rate and the X-axis is the log drug concentration. Within each graph, there are two overlaid curves, an orange one derived from luminescence measurements and a blue one derived from direct counting measurements. All of the curves across every cell line and drug pairing have highly congruent EC50 values. Additionally, while only about half of the Emax values show objective congruency, all Emax pairings are in agreement for drug effect mechanism (cytostatic, cytotoxic, anti-proliferative) except for H841/trametinib.

**Table 1.** Extracted parameters from best-fit dose–response models

Cell Line	Drug	Data Type	Hill Coef	Max Resp	EC50	Residuals
DMS114	Barasertib	Lum	8.754	−0.079	7.86E-09	9.812
DMS114	AMG-900	Lum	0.544	−0.234	1.69E-09	9.254
DMS114	TAK-901	Lum	4.181	−0.074	1.6E-08	4.990
DMS114	YM-155	Lum	9.527	−0.034	6.91E-08	13.579
DMS114	SCH-1473759	Lum	0.485	−0.131	5.74E-09	7.134
DMS114	Etoposide	Lum	14.618	−0.049	4.83E-07	16.446
DMS114	SNS-314	Lum	0.143	−0.564	2.88E-09	11.205
DMS454	Barasertib	Lum	0.192	−5.000	733	17.492
DMS454	AMG-900	Lum	2.816	−1.841	1.17	23.981
DMS454	TAK-901	Lum	0.187	−4.834	6.25E-04	61.902
DMS454	YM-155	Lum	14.273	−0.480	7.6E-08	19.917
DMS454	SCH-1473759	Lum	0.490	−5.000	3.81E-05	30.889
DMS454	Etoposide	Lum	10.439	−1.163	2.99E-06	305.999
DMS454	SNS-314	Lum	0.535	−0.511	1.44E-08	70.104
CORL279	Barasertib	Lum	0.482	0.143	5.13E-09	2.275
CORL279	AMG-900	Lum	0.047	−0.414	5.44E-09	4.109
CORL279	TAK-901	Lum	1.027	−0.114	4.42E-08	1.755
CORL279	YM-155	Lum	0.572	−0.172	2.83E-09	8.129
CORL279	SCH-1473759	Lum	0.313	−0.391	1.55E-09	4.560
CORL279	Etoposide	Lum	0.088	−0.967	5.5E-09	19.581
CORL279	SNS-314	Lum	0.000	−1.608	65.5	5.726
H1930	Barasertib	Lum	0.396	0.134	2.57E-08	4.891
H1930	AMG-900	Lum	0.950	0.500	7E-10	6.743
H1930	TAK-901	Lum	1.350	0.080	9.01E-08	6.072
H1930	YM-155	Lum	1.528	−0.253	2E-09	48.869
H1930	SCH-1473759	Lum	0.318	−0.550	5.59E-07	3.666
H1930	Etoposide	Lum	0.126	−1.089	3.37E-09	7.514
H526	Barasertib	Lum	2.818	−1.859	1.15	93.562
H526	AMG-900	Lum	2.821	−1.887	1.11	144.650
H526	TAK-901	Lum	14.720	0.402	2.39E-06	23.102
H526	YM-155	Lum	0.299	0.084	5.12E-08	9.928
H526	SCH-1473759	Lum	13.750	0.101	2.47E-06	24.866
H526	SNS-314	Lum	0.000	−0.103	2.18E-09	101.411
H1048	Barasertib	Lum	3.431	−0.209	1.44E-08	10.332
H1048	hygromycin_b	Lum	0.577	0.635	1.1E-04	13.097
H1048	Trametinib	Lum	1.054	0.238	1.37E-07	12.033
H1048	SCH-1473759	Lum	1.333	−0.219	2.86E-09	17.029
H1048	YM-155	Lum	0.995	−0.186	2.74E-09	4.925
H1048	TAK-901	Lum	2.508	−0.209	1.45E-08	17.176
H1048	SNS-314	Lum	2.507	−0.069	4.95E-09	14.051
H841	Barasertib	Lum	0.520	−0.427	3.96E-09	44.403
H841	hygromycin_b	Lum	2.817	−1.851	1.16	17.366
H841	Trametinib	Lum	0.799	−0.281	2.1E-08	17.401
H841	SCH-1473759	Lum	3.777	−0.363	4.17E-09	59.188
H841	YM-155	Lum	0.999	−0.794	9.79E-09	86.686
H841	TAK-901	Lum	2.342	−0.380	1.45E-08	13.628
H841	SNS-314	Lum	2.465	−0.377	8.42E-09	14.323
DMS53	Barasertib	Lum	2.817	−1.855	1.15	1395.696
DMS53	hygromycin_b	Lum	2.717	0.857	5.28E-08	117.582
DMS53	Trametinib	Lum	1.032	−0.659	1.38E-08	85.699
DMS53	SCH-1473759	Lum	0.201	−4.430	2.17E-03	47.346
DMS53	YM-155	Lum	0.483	−0.696	2.89E-09	46.906
DMS53	TAK-901	Lum	0.203	−5.000	4.42E-03	45.003
DMS53	SNS-314	Lum	0.781	−5.000	1.54E-04	138.641
DMS53	Vemurafenib	Lum	0.230	−5.000	5.49E-03	43.273
H1048	SNS-314	Direct	0.339	−1.673	1.31E-08	3.812
H1048	Trametinib	Direct	1.009	0.137	2.29E-08	9.877
H1048	SCH-1473759	Direct	0.601	−1.588	3.32E-09	2.783
H1048	YM-155	Direct	0.730	−1.703	3.01E-10	22.686
H1048	TAK-901	Direct	1.692	−1.294	2.11E-08	1.317
H1048	Barasertib	Direct	0.322	−2.742	6.29E-07	6.424
H841	SNS-314	Direct	0.550	−0.351	1.12E-09	1.208
H841	Trametinib	Direct	2.111	0.588	5.46E-07	0.124
H841	SCH-1473759	Direct	1.312	−0.072	5.33E-09	3.166
H841	YM-155	Direct	1.216	−0.818	2.61E-10	4.012
H841	TAK-901	Direct	2.328	−0.633	6.33E-08	0.634
H841	Barasertib	Direct	0.442	−5.000	1.55E-05	0.544

## Discussion

Here, we have outlined the development and application of a non-lytic luminescence-based assay to extract rate-based metrics of drug response. Implementation of our analysis and workflow has the potential to greatly expedite and modernize large-scale screening and characterization of drug response in a variety of disease models and culture methods. This work has traditionally been accomplished using fixed-endpoint viability metrics, which contain a significant degree of inherent biases, ultimately leading to a large potential for mischaracterization of drug effect in a variety of indices, both positive and negative. We and others have shown the value in taking continuous measurements across the duration of an experiment at multiple timepoints.^1–3,11,15,16^ However, despite the clear advantages in data quality, adoption of continuous viability assays has been relatively slow, likely due primarily to the difficulties in integrating a continuous assay into an existing setup designed for fixed-endpoint measurements. Previously, we have described the DIP rate as an unbiased metric for drug proliferation when using direct cell counting. Our analysis of continuous luminescence utilizes the same mathematical ideology, while going one step further, with a protocol that is easily adaptable to existing fixed-endpoint workflows. What this means is, by changing only the reagent preparation method and data analysis pipeline, laboratories currently setup for drug screening using a fixed-endpoint protocol could rapidly pivot to a much more quantitatively robust method with little to no adjustment of established automation. Our hope is that this additional analytical rigor at the basic science level could lead to fewer cases of therapeutic candidates failing to translate to higher order biological models.

Like any assay, NanoLuc luciferase-based continuous luminescence does have its limitations, and suffers many of the same issues surrounding MTT/MTS-based measurements such as potential overestimation of viability from active mitochondrion, and inability of use for drugs targeting redox pathways.^11,17^ These features are hardly unique to this assay, and have been generally accepted in the field for quite some time.^8,11,18–20^ Furthermore, because the assay is based upon the reducing potential of cells, the raw data is highly sensitive to the proliferation rate and the metabolic status of the cells being studied. This is to say that when comparing the raw luminescence data between cell lines, cells that proliferate quickly, have a very active metabolic status, or both, exhibit steeper luminescence trends than their slower growing or metabolically less active counterparts. However, similar to how DIP rate measurements counter proliferation rate bias involved in fixed-endpoint assays, our continuous luminescence-based analysis is not influenced by these absolute differences in the raw data, as all quantification is done within a cell line and not compared to an outside standard. This is to say that because each drug-response is calculated as a fractional value of the basal luminescence rate (for that cell line), the overall response is normalized and not amplified or diminished by the individual growth and metabolic characteristics of the line being studied. In the extreme case scenario of non-proliferating cells, we expect our proposed analysis method to remain valid, as long as the cells remain metabolically active. This example would expect to produce a relatively consistent raw luminescence trend with a slope of ~0. However, if the metabolic status of the cells being studied was not continuous (i.e. cyclically active, or non-existent) the analysis would need to be significantly adjusted and further experimentation would be required to confirm whether or not continuous luminescent measurements as described here would be the appropriate method to use at all. By structuring experiments to avoid these known factors, complex drug-response analysis can easily be simultaneously achieved across cell lines, independent of their morphology.^17^

For cell lines that are able to maintain a linear trend in luminescence for the duration of an experiment (without drug), continuous luminescence measurements offer a simple and scalable option for generating dose–response curves. This is of particular interest for cell lines that are cultured in suspension, as direct counting of suspension line cultures is not currently feasible in most situations. Additionally, because the structure of the assay is irrespective to the cell morphology, continuous luminescence allows for the quantification of cellular response across a wide range of culture characteristics such as adherent clustering, suspension aggregation, low-density culture, and slowly or non-proliferating lines. As long as the luminescent signal within the well is above the minimum threshold for the instrument, results can be obtained. Furthermore, during the course of experimentation for this method, no appreciable differences in the raw data quality were observed between cell lines exhibiting highly dense or aggregated characteristics and those exhibiting classical adherent spreading or monodisperse suspension characteristics. This suggests that the density of the culture did not play a major role in the utilization of the assay, but would need to be confirmed more thoroughly prior to implementation in three-dimensional culture settings (e.g. organoids) where diffusion of nutrients and components from the media to the center of the culture is a known issue.^21–24^ Based on the results of our experimentation, we intend to further explore the utility of NanoLuc luciferase-based luminescence by computationally modeling the dynamics of the system, potentially using luminescence rates to predict DIP rates, as well as testing its usefulness in quantifying drug-response in three-dimensional cultures (organoids). Moreover, while the mechanism of drug action (outside of redox pathway-related drugs) is not expected to influence the results of this assay, further experimentation is required using a broader library of drug classes before this can be explicitly stated. Additionally, during this process, nonsmall-molecule therapeutics (e.g. detergents, toxins, antibodies, cyclic peptides, etc.) should also be tested to assess any possible biases from therapeutic type. The results of this broader screen could also be used to assess whether induced cell death method plays any role in the utility of the assay by comparing responses to drugs which are known to induce specific cell death pathways (e.g. DNA alkylating agents for apoptosis, cytochalasin B for necrosis, SMAC mimetics combined with caspase-8 inhibition for necroptosis, etc.).^25,26^ Lastly, our most immediate goal for this work is to showcase its utility with the successful integration into a high-throughput in vitro drug screening platform.

Compared to the other currently available viability assays, continuous luminescence measures utilizing the NanoLuc luciferase signal and MT substrate offer the most advantaged and scalable platform. Unlike CellTiter-Glo, the assay is continuous and non-lytic, allowing for fewer characterization biases and the ability to use cells in downstream applications. Unlike EZ-MTT and Alamar Blue, it allows for real-time population dynamic quantification as the resulting product used in measurements (photons) is removed from the system as it is detected, instead of accumulating (formazan and resazurin), allowing for the ability to obtain negative rates. This is especially important for classifying drug effect mechanism as without real-time population dynamics it is difficult to differentiate between anti-proliferative, cytostatic, and cytotoxic drug effects. With continuous luminescence measurements, this is easily characterized, as drugs that produce rates with a value fractional to the basal rate are considered anti-proliferative, drugs that produce a rate of ~0 are considered cytostatic, and drugs that produce a negative rate are cytotoxic. Finally, unlike imaging-based methods (the only other true real-time viability assay currently available), it does not require cellular labeling or expensive imaging equipment, making it easily integrable into existing microplate-based workflows.

## Methods

### Cell lines

DMS454 and CORL-279 cells were purchased from Sigma-Aldrich (Sigma 95062832, 96020724). DMS53, DMS114, H524, H526, H841, H1048, and H1930 were purchased from the ATCC (ATCC CRL-2062, CRL-2066, CRL-5831, CRL-5811, CRL-5845, CRL-5853, CRL-5906). WM1799 cells were generously donated as a gift from the laboratory of Kim Dahlman, Ph.D. HEK293FT cells were purchased from ThermoFisher Scientific (ThermoFisher R70007).

### Cell culture

All cell lines were cultured for a minimum of 2 weeks prior to experimentation in T75 (Corning 430641 U) flasks containing appropriate media (see below) at 37°C and 5% CO_2_. Additionally, prior to any experimentation, absence of mycoplasma was confirmed using a MycoAlert Mycoplasma Detection Kit (Lonza LT07-118).

#### Appropriate media

RPMI 1640 medium (Corning 10–040-CV) supplemented with 10% FBS (Gibco 26140079) and 1% Pen-Strep (Gibco 15140122)

(CORL-279, DMS53, DMS114, DMS454, H524, H526, H1048, H1930)

DMEM/F12 medium (Gibco 11320033) supplemented with 10% FBS (Gibco 26140079), and 15 mM HEPES (Gibco 15630080)

(WM1799)

DMEM medium containing 4.5 g/L glucose (Gibco 11965092) supplemented with 10% FBS (Gibco 26140079), and 1% Pen-Strep (Gibco 15140122)

(HEK293FT)

## Static luminescence measurements

Cells were cultured for 2 weeks, spun down, and resuspended at a density of 2.86E4 cells/mL in appropriate media, NanoLuc Enzyme (Promega E499A), and MT pro-substrate (Promega G971A). Each cell line was plated on to a 384 well GreinerOne Imaging plate (Greiner 781096) at a density of 2000 cells per well serially diluted across ten wells (2000–4) with a total well volume of 70 µu\L in each. Additionally, each cell line was plated in triplicate. The plate was then incubated in a BioTek Synergy H1 at 37°C and 5% CO_2_ for 5 minutes before luminescence measurements were taken (lid on).

## Determining Linear Assay Range

Initial cell concentrations for the linearity range of the assay were determined by following the guidelines in the “Promega RealTime-Glo MT Cell Viability Assay Protocol Handbook” under subsection four, “Determining Assay Linearity for the Endpoint or Continuous-Read Format”. Briefly, cells were serially diluted and plated with RT-Glo reagents, incubated for the proposed length of experiment (120 h), while luminescence measurements were taken every 4 h. Upon completion, the luminescence trend lines were analyzed by linear regression to find a suitable cell concentration that would maintain a linear regression coefficient of >.90 for the duration of the assay (Data Not Shown).

## Continuous luminescence measurements

Cells were cultured for 2 weeks, spun down, and resuspended at a density of 4.39E3 cells/mL in appropriate media, 10 nM Sytox Green (Invitrogen S7020), NanoLuc Enzyme (Promega E499A), and MT pro-substrate (Promega G971A). Each cell line was plated on to a 384 well GreinerOne imaging plate (Greiner 781096) at a density of 300 cells per well across ten wells with a total well volume of 70 µL in each. Additionally, each cell line was plated in triplicate. The plate was then incubated in a BioTek Synergy H1 at 37°C and 5% CO_2_ for 5 min before initial luminescence and fluorescence measurements were taken (lid on). The plate was then stored at 37° Celsius and 5% CO_2_ in an incubator. Every 12 h, the plate was removed, left to equilibrate for 5 mins in the BioTek Synergy H1, and luminescence measurements were recorded. This continued for a total of 100 h, at which time the plates were discarded.

## Direct cell counting

To facilitate automated image processing, cells were engineered to express the monomeric red fluorescent protein mRuby2, integrated by dual transfection of a modified PiggyBac recombinase expressing plasmid and a custom mRuby2 containing transposon plasmid.^27,28^ Cells were seeded at 300 cells per well in 384 well GreinerOne imaging plates (Greiner 781096). DMSO (Sigma D8418) and phosphate-buffered saline (Corning 21–040-CV) were used as vehicle controls, as appropriate. Images were acquired through a 10× or 20× objective with a Cellavista HighEnd Bioimager (SynenTec Bio Services, Meunster, Germany) every 12 h as 3 × 3 or 5 × 5 montages for 120 hours. Image processing to obtain counts of cell nuclei at each timepoint was performed as previously described.^15^
